# Causality of genetically determined metabolites on anxiety disorders: a two-sample Mendelian randomization study

**DOI:** 10.1186/s12967-022-03691-2

**Published:** 2022-10-20

**Authors:** Gui Xiao, Qingnan He, Li Liu, Tingting Zhang, Mengjia Zhou, Xingxing Li, Yijun Chen, Yanyi Chen, Chunxiang Qin

**Affiliations:** 1 Department of Health Management, The Third Xiangya Hospital, Central South University, Changsha, China; 2 Xiangya Nursing School, Central South University, Changsha, China; 3 The Third Xiangya Hospital, Central South University, Changsha, China; 4 Xiangya Medicine School, Central South University, Changsha, China

**Keywords:** Anxiety disorders, Genetically determined metabolites, Mendelian randomization, Serum metabolite, 1-linoleoylglycerophosphoethanolamine

## Abstract

**Background:**

Although anxiety disorders are one of the most prevalent mental disorders, their underlying biological mechanisms have not yet been fully elucidated. In recent years, genetically determined metabolites (GDMs) have been used to reveal the biological mechanisms of mental disorders. However, this strategy has not been applied to anxiety disorders. Herein, we explored the causality of GDMs on anxiety disorders through Mendelian randomization study, with the overarching goal of unraveling the biological mechanisms.

**Methods:**

A two-sample Mendelian randomization (MR) analysis was implemented to assess the causality of GDMs on anxiety disorders. A genome-wide association study (GWAS) of 486 metabolites was used as the exposure, whereas four different GWAS datasets of anxiety disorders were the outcomes. Notably, all datasets were acquired from publicly available databases. A genetic instrumental variable (IV) was used to explore the causality between the metabolite and anxiety disorders for each metabolite. The MR Steiger filtering method was implemented to examine the causality between metabolites and anxiety disorders. The standard inverse variance weighted (IVW) method was first used for the causality analysis, followed by three additional MR methods (the MR-Egger, weighted median, and MR-PRESSO (pleiotropy residual sum and outlier) methods) for sensitivity analyses in MR analysis. MR-Egger intercept, and Cochran’s Q statistical analysis were used to evaluate possible heterogeneity and pleiotropy. Bonferroni correction was used to determine the causative association features (*P* < 1.03 × 10^–4^). Furthermore, metabolic pathways analysis was performed using the web-based MetaboAnalyst 5.0 software. All statistical analysis were performed in R software. The STROBE-MR checklist for the reporting of MR studies was used in this study.

**Results:**

In MR analysis, 85 significant causative relationship GDMs were identified. Among them, 11 metabolites were overlapped in the four different datasets of anxiety disorders. Bonferroni correction showing1-linoleoylglycerophosphoethanolamine (OR_fixed-effect IVW_ = 1.04; 95% CI 1.021–1.06; *P*_fixed-effect IVW_ = 4.3 × 10^–5^) was the most reliable causal metabolite. Our results were robust even without a single SNP because of a “leave-one-out” analysis. The MR-Egger intercept test indicated that genetic pleiotropy had no effect on the results (intercept = − 0.0013, SE = 0.0006, *P* = 0.06). No heterogeneity was detected by Cochran’s Q test (MR-Egger. Q = 7.68, *P* = 0.742; IVW. Q = 12.12, *P* = 0.436). A directionality test conducted by MR Steiger confirmed our estimation of potential causal direction (*P* < 0.001). In addition, two significant pathways, the “primary bile acid biosynthesis” pathway (*P* = 0.008) and the “valine, leucine, and isoleucine biosynthesis” pathway (*P* = 0.03*)*, were identified through metabolic pathway analysis.

**Conclusion:**

This study provides new insights into the causal effects of GDMs on anxiety disorders by integrating genomics and metabolomics. The metabolites that drive anxiety disorders may be suited to serve as biomarkers and also will help to unravel the biological mechanisms of anxiety disorders.

**Supplementary Information:**

The online version contains supplementary material available at 10.1186/s12967-022-03691-2.

## Introduction

Anxiety disorders are a significant health problem widespread and are the leading psychiatric causes of the global burden of diseases [1]. The World Health Organization (WHO) has also ranked anxiety disorders as one of the largest causes of disability worldwide largely due to their high prevalence, chronicity, and comorbidity [2, 3]. Effective prevention and treatment of anxiety disorders are critical to reduce the morbidity and disability. Notably, the exploration of the biological mechanism is the basis for the prevention and therapy of anxiety disorders [4]. Multiple factors, such as psychological and genetic factors, are thought to be involved in the biological mechanisms of anxiety disorders [4, 5]. However, anxiety disorders are complex conditions, and their biological mechanisms are not fully understood. Although progress in genetics (particularly genome-wide association studies (GWASs)) have largely improved the development of etiology research for mental disorders [6–8], there is still a great barrier translating these genetic findings into biological mechanisms.

In recent years, modern omics-based technologies, including metabolomics, have made a positive contribution to the exploration of disease mechanisms. Specifically, metabolomics can provide novel information into the biological mechanisms of diseases by revealing the intermediate metabolites and altered metabolic pathways [9, 10]. A recent robust study of GWAS of metabolites has identified the disease-relevant loci and suggest mechanisms for diseases and disease-related traits [11]. Several studies have suggested that metabolites are functional intermediates that can be used to illustrate the potential biological mechanisms related to the genetics of mental disorders [12–14]. It is worth noting that metabolites are the final products or the intermediate of metabolism that can play important in human. The database of genotype dependent metabolic phenotypes (also known as genetically determined metabolites (GDMs)) has recently been established using a GWAS involving non-targeted metabolomics [15, 16]. The developed GDMs can promote insight of the underlying relationship of human serum metabolites and associative genetic variants in the biological mechanisms of mental disorders by providing functional intermediates [17–19]. Studies have shown the significance of GDMs in the biological mechanism of major depression, bipolar disorders, autism spectrum disorders and hyperactivity disorders [17]. However, GDMs and pathway analysis geared toward exploring the biological mechanisms of anxiety disorders are still lacking, which calls for a deep analysis to determine the role played by the effects between genetic variation and metabolites in the biological mechanisms of anxiety disorders.

Mendelian randomization (MR) analysis is a useful epidemiological research strategy in which genetic variants are used to connect exposure with outcome as instrumental variables (IV) for assessing causal relationships. Compared to other epidemiological research strategies, MR can provide unbiased estimates on how genotypes are decided at conception, and are commonly not susceptible to confounding factors and reverse causation [20]. Given this huge advantage, MR has been widely applied in the past decade to infer causality of related risk exposure to disease using publicly available GWAS summary statistics [21, 22]. Recently, GWAS have extended the metabolic spectrum, from which an atlas of GDMs was developed.

Herein, we speculated that this GDMs atlas could be used to infer the causality of GDMs on anxiety disorders. Consequently, we implemented a two-sample MR approach to: (1) assess the causal effects of human serum metabolites on anxiety disorders; (2) identify the GDMs that have causal effects on four different GWASs of anxiety disorders; and (3) identify potential metabolic pathways which might help to understand the mechanism of anxiety disorders.

## Materials and methods

### MR design and data source

The general design of this MR research was stated in Additional file 1: Fig. S1. The study methods were compliant with the STROBE-MR checklist [23]. Genetic association data for serum metabolites were obtained from the metabolomics GWAS server (http://metabolomics.helmholtz-muenchen.de/gwas/). Notably, Shin et al. [24] reported the most comprehensive exploration of genetic influences on human metabolism so far, by performing a GWAS of non-targeted metabolomics, which successfully screened out 486 metabolites with genetic influences on human serum metabolites. In more detail, a total of 7824 participants were recruited from two European population cohorts, including 1768 participants from the KORA F4 study in Germany and 6056 from the UK Twin Study. Both studies were approved by local ethics committees, and all participants voluntarily signed informed consent before the study. Fasting serum was analyzed using non-targeted mass spectrometry analysis. For metabolic analyses, standardized processes of identification and relative qualification were conducted using Metabolon, Inc. (https://www.metabolon.com/). After strict quality control, 486 metabolites in total were analyzed, including 309 known metabolites and 177 unknown metabolites. Moreover, the 309 known metabolites were further classified into eight biochemical classes (peptides, nucleotides, amino acids, energy, cofactors and vitamins, lipids, carbohydrates, and xenobiotics) in conformity to the Kyoto Encyclopedia of Genes and Genomes (KEGG) database. Notably, genotyping information for the two cohorts are described detailly in previous research [24, 25]. Finally, there were approximately 2.1 million single nucleotide polymorphisms (SNPs) in the GWAS meta-analysis.

### Selection of instrumental variables (IVs)

In principle, the instrumental variables used in MR analysis must satisfy the following three assumptions: (1) IVs must be relevant to exposure (i.e.,: metabolites); (2) IVs must be associated with outcome (i.e., anxiety disorders) only via exposure (i.e., metabolites); and (3) IVs must be independent of any confounder [26]. To determine the IVs for the 486 metabolites, some procedures were done to ensure that the first assumption was true. First, the genetic variants were extracted with association thresholds at *P* < 1 × 10^–5^, which are mostly used in MR analysis to elucidate a greater variation when few SNPs are available for exposure. Second, independent variants were identified using a clumping procedure implemented in R software, in which a linkage-disequilibrium threshold of r^2^ < 0.5 within a 5000 kb window in the European 1000 Genomes Project Phase 3 reference panel was set. Instrumental SNPs were selected by removing palindromic SNPs with middle allele frequency (MAF). Palindromic SNPs are those with the A/T or G/C allele, whereas the MAF is from 0.01 to 0.30. SNPs with the incorrect causal direction are excluded by MR Steiger filters. SNPs with an MAF less than 0.01 were also excluded from the original GWAS because of their low confidence level. Next, we used the explained variance (*R*^*2*^) and *F* statistic parameters to determine whether the identified IVs were powerful enough to represent metabolite levels. The above design formula is presented in Additional file 2: Table S1. Typically, a threshold of *F* > 10 is suggested for MR analysis. The mRnd was used to calculate the statistical power for Mendelian randomization (https://cnsgenomics.shinyapps.io/mRnd/).

### GWAS of anxiety disorders

This study aimed at evaluating the potential causal relationship between metabolites and anxiety disorders with a wide range of impact in both males and females from MRC-IEU. Anxiety disorders are typically diagnosed on the basis of structured clinical interviews for the fifth edition of the Diagnostic and Statistical Manual of Mental Disorders (DSM-5) and the tenth edition of the International Classification of Diseases (ICD-10). Anxiety disorders also can be screened with self-report questionnaires. Moreover, providing care for mental health problems including anxiety disorders concerns general practitioners, and psychiatrists [27, 28]. Therefore, given the completeness of the data and diagnosis of anxiety disorders, we analyzed the data obtained from different sources, including patients diagnosed by psychiatrists for anxiety (53,414 cases and 407,288 controls); unspecified anxiety disorders diagnosed by secondary ICD-10 (1523 cases and 461,487 controls); patients diagnosed by general practitioners for anxiety (158,565 cases and 300,995 controls); and self-reported anxiety (6410 cases and 462,933 controls). Additional file 2: Table S2 shows the detailed information. Notably, all IVs were extracted from MR-Base (http://app.mrbase.org/) database (20).

### MR analyses

The IVW method (when there is heterogeneity, a random effect model of IVW is used, and if there is no heterogeneity, a fixed effect model of IVW is used) was used to assess causal effects for two-sample MR analyses. Notably, the IVW method can provide a consistent assessment of the causality of the exposure when each variant satisfies all three assumptions of valid instrumental variables. An estimate of IVW can be obtained by calculating the slope of the weighted linear regression [26]. Next, sensitivity analysis was performed using MR-Egger approach, which can provide consistent estimates with invalid instruments [29]. The MR-Egger can discover the violations of the IVs assumption and provide estimates of effects unaffected by these violations. Moreover, the weighted median method also provided consistent estimates, with up to 50% of the variants being thought noneffective instruments. MR-PRESSO is another novel MR method that can check and rectify horizontal pleiotropic outliers, thereby providing a right estimate [30]. To assess the possibility of horizontal pleiotropy and bias caused by ineffective IVs, MR-Egger intercepts were also calculated [31]. Moreover, this study employed the MR-PRESSO global test to assess the existence of horizontal pleiotropy. We calculated the odds ratios (OR) to measure causal effects, as well as Cochran’s Q statistics, to estimate heterogeneity among SNPs [32]. A “leave-one-out” sensitivity analysis was implemented to determine whether results were affected by a single SNP [31]. Additionally, we performed the MR Steiger directionality test to ascertain whether our results supported our hypothesis. Notably, all analyses were conducted using Two Sample MR 0.5.6 and MR-PRESSO packages in R software (version 3.6.0). *P* < *0.05* was considered statistically significant. We adopted a multiple-testing-adjusted threshold of *P* < 1.03 × 10^−4^ (0.05/486) using the Bonferroni correction to declare a statistically significant, causal relationship [33]. We also reported metabolites that had a *P* < 0.05, but were above the Bonferroni-corrected threshold, as suggestive risk predictors for anxiety disorders.

### Metabolic pathway analysis

Metabolic pathway analysis was performed using the web-based Metaconflict 5.0. (https://www.metaboanalyst.ca/) [34]. Functional enrichment analyses and the pathway analyses module were used to identify underlying metabolite groups or pathways which may be relevant to the biological process of anxiety disorders. In all, 25 serum metabolic pathways were screened out from two metabolite databases, including 24 from both the Small Molecule Pathway Database (SMPD) [35] and the KEGG database, and one from SMPD alone. Notably, this study only analyzed the metabolites that passed the advised threshold of association by IVW (*P*_IVW_ < 0.05).

## Results

### Strength of the instrumental variables

We performed a two-sample MR analysis to evaluate the causality of GDMs on anxiety disorders using four different pairs of GWAS summary data. The generated IVs of 486 metabolites are ranging from 3 to 56 SNPs (glycodeoxycholate generated the least IVs: 3 SNPs; and p-acetamidophenylglucuronide generated the most IVs: 56 SNPs); These generated IVs could explain 0.011–0.225% of the variance of their corresponding metabolites (Additional file 2: Tables S3–S11). In addition, the minimum *F* statistic of these IVs was 10.23, suggesting that all IVs were sufficiently effective for the MR analysis of the 486 metabolites (*F* statistic > 10).

### Causality of genetically determined metabolites on anxiety disorders

**Fig. 1:**
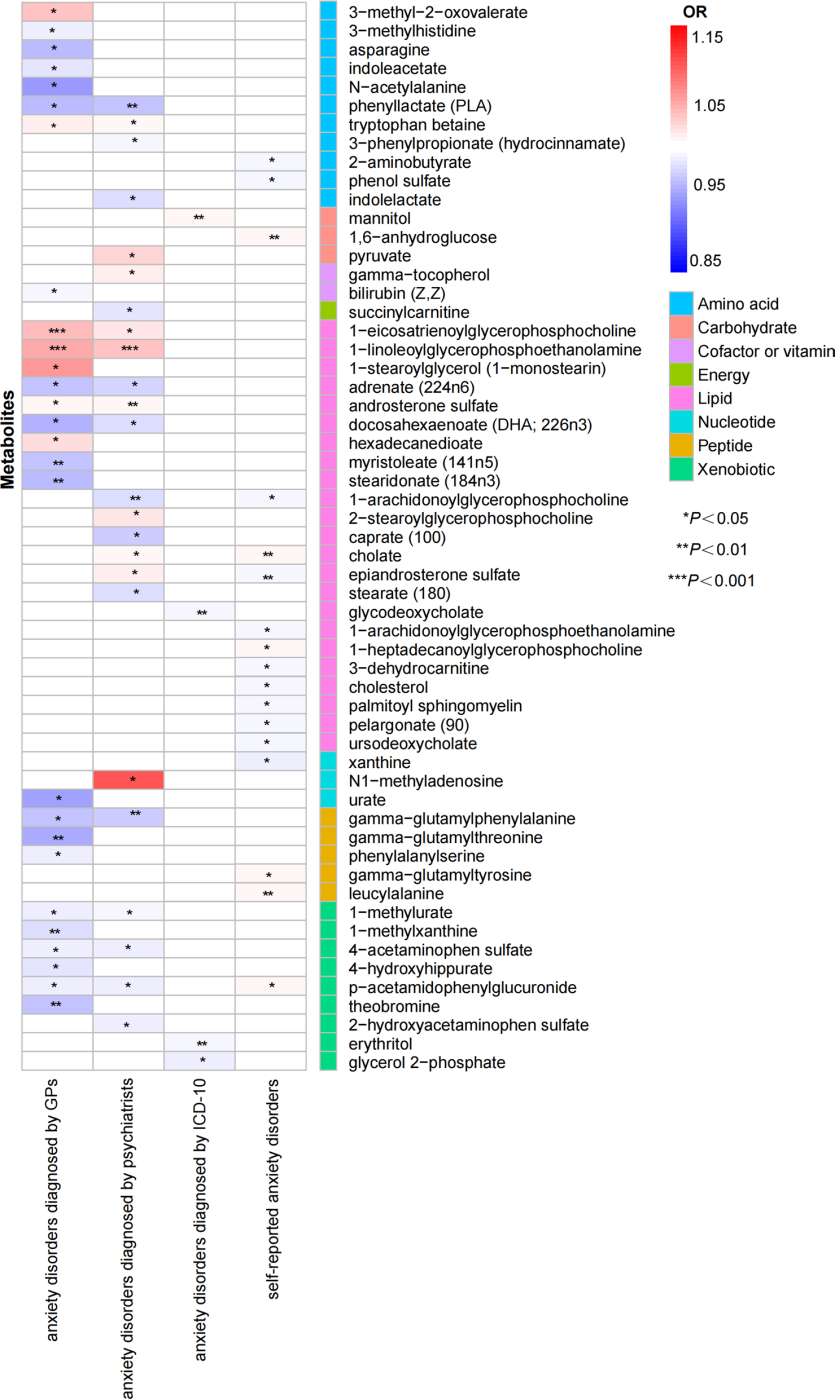
Mendelian randomization associations of known metabolites on the risk of the four different GWAS datasets of anxiety disorders (derived from the fixed-effect IVW analysis). IVW, inverse-variance weighted

The IVW method was employed to confirm the causality among the 486 metabolites and anxiety disorders using four pairs of GWAS summary data. 103 remarkable causative association features (matching with 85 unique metabolites) were conformed at *P*_IVW_ < 0.05 in total, including 57 known metabolites and 28 unknown metabolites. Additional file 2: Table S12 shows the known metabolites that were significantly associated with the GWAS datasets of anxiety disorders. Additional file 2: Table S13 shows the characteristics of SNPs and their genetic associations with known metabolites and the anxiety disorders. And Fig. 1 shows the known metabolites significantly associated with anxiety disorders (as well as the subgroup analysis of metabolites significantly associated with the four different GWAS datasets of anxiety disorders). Table 1 shows that there were 11 overlapped metabolites in the four different GWAS datasets of anxiety disorders (there were no metabolites that overlapped with the other three anxiety disorders GWAS in the unspecified anxiety disorders diagnosed by secondary ICD-10). It is worth noting that there may be some common molecular mechanisms between four different GWAS datasets of anxiety disorders. Next, we performed the Bonferroni correction to determine the causative association features (*P* < 1.03 × 10^–4^). Results found one causal effect feature of metabolites associated with anxiety disorders diagnosed by psychiatrists, namely 1-linoleoylglycerophosphoethanolamine (*P*_fixed-effect IVW_ = 4.31 × 10^–5^) (Additional file 2: Table S14). The statistical power of 1-linoleoylglycerophosphoethanolamine for anxiety diagnosed by psychiatrists was 47%. In particular, fixed-effect IVW estimates demonstrated that 1-linoleoylglycerophosphoethanolamine increased the risk of anxiety disorders (OR 1.04; 95% CI 1.021–1.06; *P*_fixed-effect IVW_ = 4.3 × 10^–5^) (Table 1). Moreover, as for the additional methods, weighted median analysis (OR 1.049, 95% CI 1.022–1.077, *P* = 0.0003), and MR-Egger analysis (OR 1.092, 95% CI 1.040–1.147, *P* = 0.005) indicated consistent results (Additional file 2: Table S7). As indicated by the MR Steiger directionality test results, our estimate of causal direction was accurate (*P* < 0.001). In addition, we performed a reverse MR analysis, which found no causal relationship between anxiety disorders (exposure) and 1-linoleoylglycerophosphoethanolamine (outcome) (Additional file 2: Table S15); Finally, to verify our results, we performed MR analysis with two additional GWAS data of anxiety disorders (finn-b-KRA_PSY_ANXIETY: 20,992 cases and 197,800 controls; finn-b-KRA_PSY_ANXIETY_EXMORE: 20,992 cases and 166,584 controls), and also found that the 1-linoleoylglycerophosphoethanolamine might have causality with anxiety disorders (Additional file 2: Table S16). Consequently, we discovered that the 1-linoleoylglycerophosphoethanolamine might be causally associated with anxiety disorders, and the result is reliable.

**Table 1:** Overlapped metabolites identified for the four different GWAS datasets of anxiety disorders

Description	Anxiety disorders diagnosed by psychiatrists		Unspecified anxiety disorders diagnosed by secondary ICD-10		Anxiety disorders diagnosed by general practitioners		Self-reported anxiety	
	OR (95% CI)	*P*	OR (95% CI)	*P*	OR (95% CI)	*P*	OR (95% CI)	*P*
1-arachidonoylglycerophosphocholine	0.975(0.958–0.992)	0.004**					0.993(0.987–0.999)	0.020
1-eicosatrienoylglycerophosphocholine	1.017(1.001–0.033)	0.039*			1.045(1.022–1.069)	0.000***		
1-linoleoylglycerophosphoethanolamine	1.040(1.021–1.060)	0.000***			1.054(1.025–1.084)	0.000***		
adrenate (224n6)	0.969(0.942–0.997)	0.032*			0.958(0.920–0.997)	0.035*		
androsterone sulfate	1.010(1.003–1.016)	0.007**			1.009(1.001–1.017)	0.031*		
cholate	1.009(1.002–1.017)	0.011*					1.004(1.001–1.007)	0.005
docosahexaenoate (DHA; 226n3)	0.971(0.947–0.995)	0.019*			0.949(0.906–0.994)	0.025*		
epiandrosterone sulfate	1.012(1.001–1.023)	0.029*					0.995(0.992–0.998)	0.003
gamma-glutamylphenylalanine	0.961(0.935–0.987)	0.003**			0.959(0.921–0.999)	0.043*		
phenyllactate (PLA)	0.958(0.933–0.984)	0.002**			0.952(0.907–0.998)	0.041*		
tryptophan betaine	1.008(1.002–1.015)	0.010*			1.014(1.003–1.025)	0.012*		

OR, odds ratio; CI, confidence interval

*p < 0.05

**p < 0.01

***p < 0.001

### Sensitivity analyses

**Fig. 2:**
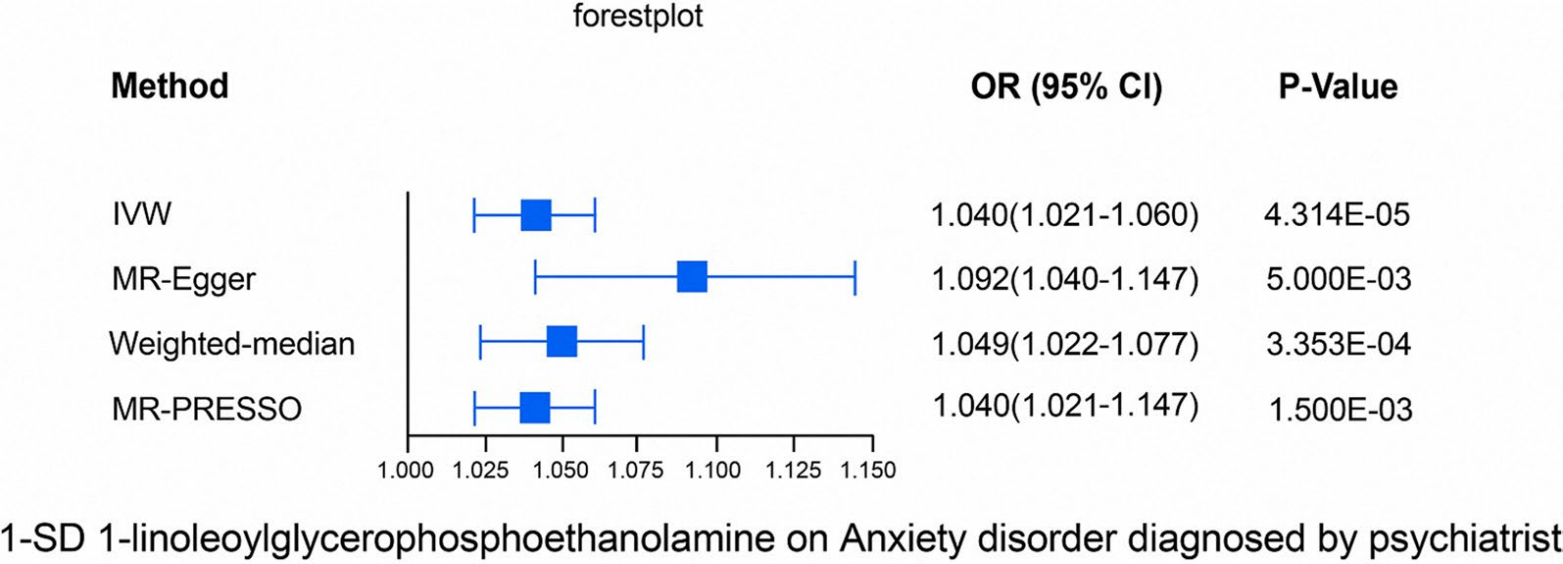
Sensitivity analysis for1-linoleoylglycerophosphoethanolamine on the anxiety disorders diagnosed by psychiatrists passing Bonferroni correction

While the IVW method is extremely effective for inferring causality between an exposure and a disease outcome, it is known to be susceptible to weak instrument bias. Therefore, we further conducted sensitivity and pleiotropy analysis to assess the robustness of the causality. The sensitivity analyses result for 1-linoleoylglycerophosphoethanolamine on the anxiety disorders diagnosed by psychiatrists are shown in Fig. 2. Notably, the causal relationship was reliable if three extra MR tests were performed, with results showing that there was no proof of horizontal pleiotropy for the 1-linoleoylglycerophosphoethanolamine presented in Additional file 1: Figs. S2, S3. And based on the results of “leave-one-out” method, MR analysis was responsible, and single SNPs did not affect the results (Additional file 1: Fig. S4). Moreover, for the IVW method, Cochran's Q statistic was 12.12 (*P* = 0.436). The Cochran's Q statistic was 7.68 (*P* = 0.742) for the MR-Egger method. The results of Cochran's Q test suggested little heterogeneity. MR-Egger regression was also conducted to examine the horizontal pleiotropy between IVs and results, but no remarkable intercept was discovered (intercept = − 0.0013, SE = 0.0006, *P* = 0.06). Furthermore, MR-PRESSO results indicated no horizontal pleiotropy in the MR study (*P* = 0.42). A funnel plot (Additional file 1: Fig. S5) displays neither horizontal pleiotropy nor heterogeneity in our MR study. All the results shown that the causal effect of 1-linoleoylglycerophosphoethanolamine on the anxiety disorders diagnosed by psychiatrists appears to be reliable. Furthermore, Table 2 shows that five association metabolites passed all the sensitivity analyses (*P* < 0.05) without horizontal pleiotropy in the 11 overlapped metabolites. MR-Egger regression was also conducted to examine the horizontal pleiotropy of the five association metabolites, no remarkable intercept was discovered. And the results of Cochran's Q test suggested little heterogeneity (Table 2). The genetic variants that explain the relationship between the five metabolites and anxiety disorders are presented in Additional file 2: Tables S17–S21 and Additional file 1: Figs. S6–S9, separately. Moreover, we performed the reverse MR analysis, which found no causal relationship between anxiety disorders (exposure) and the five association metabolites (outcome) (Additional file 2: Table S15). The *P*-value distribution of the *P*_fixed-effect_ IVW < 0.05 metabolites on anxiety disorders are presented in Additional file 1: Fig. S10.

**Table 2:** five association metabolites passed all the sensitivity analyses

Description	*P*_fixed-effect IVW_	*P*_MR-Egger_	*P*_Weighted-median_	*P*_MR-PRESSO_	*P*_MR-PRESSO Global_	*P*_MR-Egger intercept_	*P*_IVW. Q_
1-arachidonoylglycerophosphocholine for anxiety disorders diagnosed by psychiatrists	0.0042**	0.026*	0.0002***	0.0096**	0.149	0.338	0.207
androsterone sulfate for anxiety disorders diagnosed by psychiatrists	0.0065**	0.0185*	7.63 × 10^–5***^	0.0136*	0.113	0.311	0.091
1-linoleoylglycerophosphoethanolamine for anxiety disorders diagnosed by general practitioners	0.002**	0.0148*	0.0001***	0.003**	0.366	0.146	0.408
1-eicosatrienoylglycerophosphocholine for anxiety disorders diagnosed by general practitioners	0.001**	0.0447*	0.116	0.0005***	0.696	0.440	0.670
epiandrosterone sulfate for self-reported anxiety	0.0028**	0.023*	0.0027**	0.007**	0.554	0.249	0.620

*p < 0.05

**p < 0.01

***p < 0.001;

### Metabolic pathway analyses

Two important metabolic pathways which mainly involved in the anxiety disorders were identified in the metabolic pathways analyses (Additional file 2: Table S22). Results showed that the “primary bile acid biosynthesis” pathway might be relevant to the development of self-reported anxiety disorders (*P* = 0.008), whereas “valine, leucine, and isoleucine biosynthesis” pathway was found to be associated with anxiety disorders diagnosed by general practitioners (*P* = 0.03).

## Discussion

This Mendelian randomization study provides unbiased evaluation of the causal relationship between GDMs and anxiety disorders using four different GWAS datasets, including anxiety disorders diagnosed by psychiatrists, unspecified anxiety disorders diagnosed by secondary ICD-10, anxiety diagnosed by general practitioners, and self-reported anxiety disorders. We identified 85 metabolites relevant to the risk of anxiety disorders after using genetic variants as probes. Among them, 11 metabolites were overlapped in the four different datasets of anxiety disorders, with Bonferroni correction showing that 1-linoleoylglycerophosphoethanolamine had the most reliable causal relationship. Moreover, pathway enrichment analysis identified two significant metabolic pathways, the “primary bile acid biosynthesis” pathway and the “valine, leucine, and isoleucine biosynthesis” pathway, which are mainly involved in the anxiety disorders.

As far as we know, this is the first MR study that has combined genomics with metabolomics to evaluate the causality of GDMs on anxiety disorders. Herein, results identified a cluster of metabolites in serum showing relationship with anxiety disorders, among which 1-linoleoylglycerophosphoethanolamine had a robust effect on anxiety disorders diagnosed by psychiatrists and anxiety disorders diagnosed by general practitioners. In a previous study, researchers conducted a delivering preterm with or without preeclampsia population-based birth cohort, and found that higher 1-linoleoylglycerophosphoethanolamine density decreased odds of preeclampsia [36]. The result is corresponding with previous research which revealed that women who developed preeclampsia had lower levels of lysophospholipids compared to healthy term pregnancies [37–39]. In addition, preeclampsia women were more likely to be diagnosed with anxiety disorders. 1-linoleoylglycerophosphoethanolamine is an important member of the phosphatidylethanolamine (PE) family, whose components mainly include fatty acids, ethanolamine, phosphoric acid, and glycerol [40]. As a lipid chaperone, PE can assist in the folding of certain membrane proteins, and is closely associated with anxiety disorders. A previous study found that stress-prone Wistar-Kyoto rats had lower PE in the anxiety state compared to the non-stress prone rats, suggesting that PE may be associated with the anxiety disorders [41]. Studies have also found that alcohol-dependent patients are often accompanied by anxiety disorders when they quit drinking, and the concentration of PE in plasma is higher after alcohol-dependent patients quit drinking [42]. Notably, Yang et al. [17] explored the relationship between metabolism and some psychiatric disorders, and found that the genetic associations of 1-linoleoylglycerophosphoethanolamine were involved in the risk of major depression. Clinically, the comorbidity of depression and anxiety is common, and the two often influence each other. Although 1-linoleoylglycerophosphoethanolamine seems like a hopeful biomarker for anxiety disorders, further studies should be conducted to clarify the relevant mechanism.

This MR analysis also identified certain metabolites, some of which had been reported in previous research. Androsterone sulfate whose chemical formula is 3α-hydroxy-5α-androstan-17-one 3α-sulfate, is one of the primary urinary metabolites of androgens. Previous studies reported that metabolite networks enriched in androsterone sulfate, tyrosine, indoxyl sulfate, or caffeine are associated with a negative personality [43]. Negative personality manifested as inhibition in social situations is often described as distress or anxiety. Moreover, sleep-related impairment was inversely correlated with androgen deprivation therapy-induced reduction in androsterone sulfate [44]. Given that people with sleep disorders are often accompanied by anxiety disorders, androsterone sulfate might play a role in neurodevelopment of anxiety disorders. Epiandrosterone sulfate is a vital endogenous androstane steroid produced by the adrenal cortex. Notably, the steroid is an important neurosteroid and neurotrophin, which plays an important physiological function in human body. A recent omics investigation into chronic widespread musculoskeletal pain revealed that epiandrosterone sulfate is an important biomarker [45]. Considering that people with chronic pain are often accompanied by anxiety disorders, epiandrosterone sulfate might play a role in neurodevelopment of anxiety disorders. In addition, p-acetamidophenylglucuronide showed robust association with intelligence, and thus it can be used to predict important health outcomes that may affect anxiety disorders [15]. It is worth mentioning that our results are consistent with above results and emphasize the significance of genetics in the progression of mental disorders. It is worth noting that the use of drugs also has an effect on metabolite profiles. The baseline characteristics and the drug use of the participants involving in the study are listed in Additional file 2: Table S23–S25. The effect of drugs (such as antihypertensive and lipid lowering drugs) on the human metabolite concentrations is still not fully understood, and further studies are needed.

In this study, the metabolic pathway analysis showed that “primary bile acid biosynthesis”, and “valine, leucine, and isoleucine biosynthesis” pathways are mainly associated with anxiety disorders. Notably, primary bile acids are synthesized in liver cells by cytochrome P450-mediated oxidation of cholesterol, resulting in the synthesis of primary bile acids (such as deoxycholic acids). Primary bile acids have rich functions and are important physiological factors for intestinal nutrient absorption, bile secretion of lipids, toxic metabolites and exotic organisms. At the same time, primary bile acids are signaling molecules and metabolic regulators, which play an important role in activating nuclear receptor and G-protein-coupled receptor (GPCR) signals, regulating liver lipid, glucose and energy homeostasis and maintaining metabolic homeostasis. Studies have proved that primary bile acid metabolites/pathways are involved in several aspects of brain function and behavior [46]. Moreover, it has been reported that changes in the gut microbiota composition may be associated with alterations in the primary bile acid metabolism that are involved in the biological process of psychological disorders in Crohn’s disease [47]. A traditional Chinese medicine cohort revealed that amino acid metabolism, such as cysteine and methionine metabolism, might be involved in brain health disorders characterized by alterations in evaluation of bile acid biosynthesis [48]. Altogether, these findings suggest that biosynthesis of primary bile acids might play an important role in the biological mechanism of anxiety disorders.

Valine, leucine, and isoleucine are structurally associated with branched-chain fatty acids and are important members of the family of 9 essential amino acids. Metabolism of valine, leucine and isoleucine have been proved to be associated with cancer progression in numerous studies, and key proteins in metabolic pathways may act as potentially prognostic and diagnostic biomarkers in human cancers [49]. Given that people with cancers are often accompanied by anxiety disorders, valine, leucine, and isoleucine might play a role in neurodevelopment of anxiety disorders. Previous studies have proved that plasma concentrations of valine, leucine, and isoleucine are increased significantly in conditions associated with insulin resistance, such as obesity and diabetes mellitus [50]. Research has demonstrated that anxiety symptoms are prevalent in people with diabetes, and may affect diabetes management and glycemic control [51, 52]. Chen et al. [53] established a rat model to explore the potential mechanisms of antidepressant effects, with the mainly enriched pathways being valine, leucine, and isoleucine degradation. It should be noted that this model is built on liver tissue, suggesting that the liver may potentially be connected with mental disease through some metabolites. To sum up, the biosynthesis of valine, leucine, and isoleucine might be relevant to the biological mechanism of anxiety disorders.

However, this study had several limitations. First, given the classification of the original data, we could not further subdivide the pressure type of anxiety disorders in combination with the ICD classification standard, and thus we could only analyze the anxiety disorders as a whole. Second, the power of the IVs depends largely on the sample size of GWASs, therefore, more data are needed to improve the accuracy of the generated GDMs. Third, although Mendelian randomization has been shown to be a powerful method to assess the causality between human blood metabolites and anxiety disorders, the results should be verified by further studies based on experimental data. Fourth, the veracity of the MR analysis relies largely on the explanation of the instrumental variables on exposure. Therefore, it is necessary to expand the sample size to provide a more accurate assessment of the genetic impact on metabolites. Fifth, due to insufficient data, we used metabolites with uncorrected P values for pathway analysis. At last, although this study identified multiple metabolites that contribute to the risk of anxiety disorders, further studies are needed to reveal their roles in the pathogenic mechanism of anxiety disorders.

## Conclusion

In conclusion, this MR research totally identified 85 metabolites that may have causality on the pathogenesis of anxiety disorders, including 11 known metabolites having causality on more than one type of anxiety disorders. And 1-linoleoylglycerophosphoethanolamine had a robust effect on anxiety disorders among them. Moreover, this study identified two important metabolic pathways that may be relevant to the pathology of anxiety disorders. Collectively, our findings will provide valuable insights on using some metabolites as potential biomarkers to explore the targeted drugs for treating human diseases, but more studies are required to validate the results.

## Supplementary Information

**Additional file 1**: **Figure S1.** The rationale of Mendelian randomization; **Figure S2.** Scatter plots of the causal association of 1-linoleoylglycerophosphoethanolamine on the risk of anxiety disorders diagnosed by psychiatrists. **Figure S3.** Forest plots for 1-linoleoylglycerophosphoethanolamine on anxiety disorders diagnosed by psychiatrists; **Figure S4.** Leave-one-out plots for 1-linoleoylglycerophosphoethanolamine on anxiety disorders diagnosed by psychiatrists; **Figure S5.** Funnel plots for 1-linoleoylglycerophosphoethanolamine on anxiety disorders diagnosed by psychiatrists; **Figure S6.** Scatter plots of the genetic association of six metabolites on the risk of anxiety disorders. **Figure S7.** Forest plots for six potential metabolites on anxiety disorders; **Figure S8.** Leave-one-out plots for the six potential metabolites on anxiety disorders; **Figure S9.** Funnel plots for six potential metabolites on anxiety disorders; **Figure S10.** The p-value distribution of the P_fixed IVW_ < 0.05 metabolites on anxiety disorder.

**Additional file 2: Table S1.** The formula used to calculate R^2^ and F statistic between exposure and outcome; **Table S2.** Summary statistics of the anxiety disorders; **Table S3–S12.** Mendelian Randomization estimation for Amino acid(Table S3); Carbohydrate (Table S4); Co-factor or vitamin(Table S5);Energy(Table S6); Lipid(Table S7); Nucleotide (Table S8); Peptide (Table S9); Xenobiotic(Table S10);X(unknow metabolites) (Table S11);known metabolites (Table S12) on the risk of anxiety disorders; **Table S13.** The characteristics of SNPs and their genetic associations with known metabolites and the anxiety disorders; **Table S15.** The reverse MR analysis of 1-linoleoylglycerophosphoethanolamine and anxiety disorders using various methods; **Table S16.** Association of 1linoleoylglycerophosphoethanolamine and additional anxiety disorders GWAS using various methods; **Table S14, S17**–S**21.** Summary statistics for the associations of the five metabolites (passed all the sensitivity analyses)-associated SNPs with these exposures and the anxiety disorders from MRC-IEU Consortium; **Table S22.** Metabolic Pathway Associated with self-reported anxiety; **Table S23.** Clinical characteristics of participants involved in the study; **Table S24.** Characteristics of the KORA population related to antihypertensives and lipid-lowering drugs; **Table S25.** Characteristics and patterns of anti-hyperglycemic treatment of participants with self-reported type 2 diabetes in the KORA F4 study.

## Data Availability

Publicly available datasets were analyzed in this study. This data can be found here: (http://metabolomics.helmholtz-muenchen.de/gwas/) and (http://app.mrbase.org/).

## Abbreviations

GDMs: Genetically determined metabolites
MR: Mendelian randomization
GWAS: Genome wide association study
IV: Instrumental variable
MR-PRESSO: MR pleiotropy residual sum and outlier
IVW: Inverse-variance weighted
KEGG: Kyoto encyclopedia of genes and genomes
SNPs: Single nucleotide polymorphisms
SMPD: Small molecule pathway database
WHO: World health organization
DSM-5: The fifth edition of the diagnostic and statistical manual of mental disorders
ICD-10: The tenth edition of the international classification of diseases
PE: Phosphatidylethanolamine
