# Supplementary material for: Causality of genetically determined metabolites on anxiety disorders: a two-sample Mendelian randomization study
Source: J Transl Med. 2022 Oct 20;20:475. doi: 10.1186/s12967-022-03691-2 (PMC9583573; doi:10.1186/s12967-022-03691-2)
Supplement: Supplementary file 1 — Additional file 1: Figure S1. The rationale of Mendelian randomization; Figure S2. Scatter plots of the causal association of 1-linoleoylglycerophosphoethanolamine on the risk of anxiety disorders diagnosed by psychiatrists. Figure S3. Forest plots for 1-linoleoylglycerophosphoethanolamine on anxiety disorders diagnosed by psychiatrists; Figure S4. Leave-one-out plots for 1-linoleoylglycerophosphoethanolamine on anxiety disorders diagnosed by psychiatrists; Figure S5. Funnel plots for 1-linoleoylglycerophosphoethanolamine on anxiety disorders diagnosed by psychiatrists; Figure S6. Scatter plots of the genetic association of six metabolites on the risk of anxiety disorders. Figure S7. Forest plots for six potential metabolites on anxiety disorders; Figure S8. Leave-one-out plots for the six potential metabolites on anxiety disorders; Figure S9. Funnel plots for six potential metabolites on anxiety disorders; Figure S10. The p-value distribution of the Pfixed IVW < 0.05 metabolites on anxiety disorder. [file 12967_2022_3691_MOESM1_ESM.docx]

Additional Material

## Additional Figures


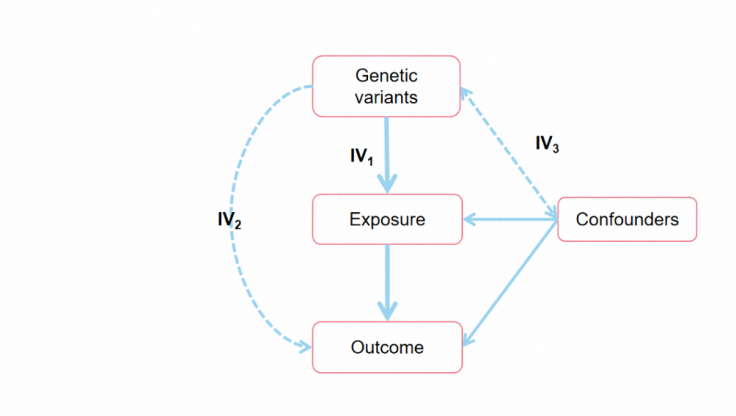


**Figure S1.** The rationale of Mendelian randomization.

IV assumption 1 represents the instrumental variables(IVs)are strongly associated with the exposure;IV assumption 2 indicates the IVs must influence the outcome only through the exposure; IV assumption 3 shows the IVs must not associate with con-founders.

**
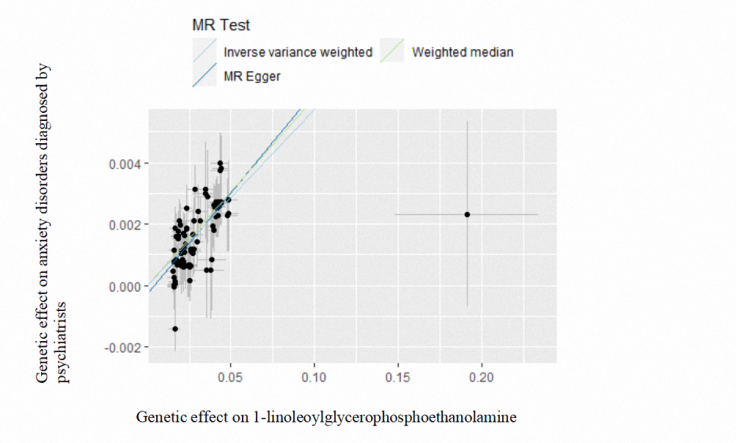
**

**Figure S2:** Scatter plots of the causal association of 1-linoleoylglycerophosphoethanolamine on the risk of anxiety disorders diagnosed by psychiatrists.


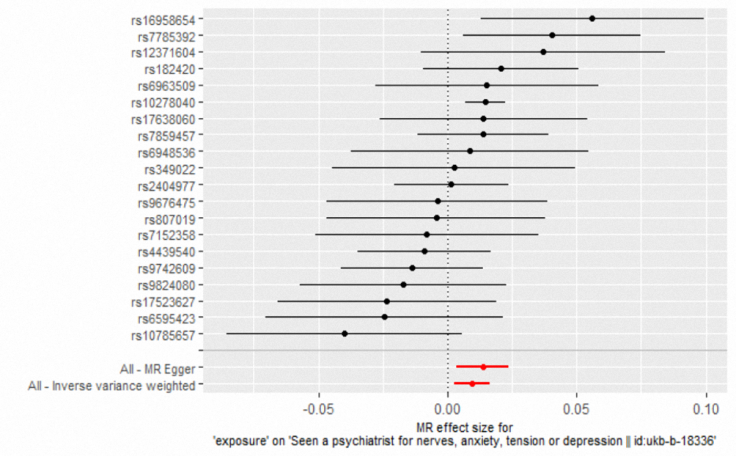


**Figure S3:** Forest plots for 1-linoleoylglycerophosphoethanolamine on anxiety disorders diagnosed by psychiatrists.


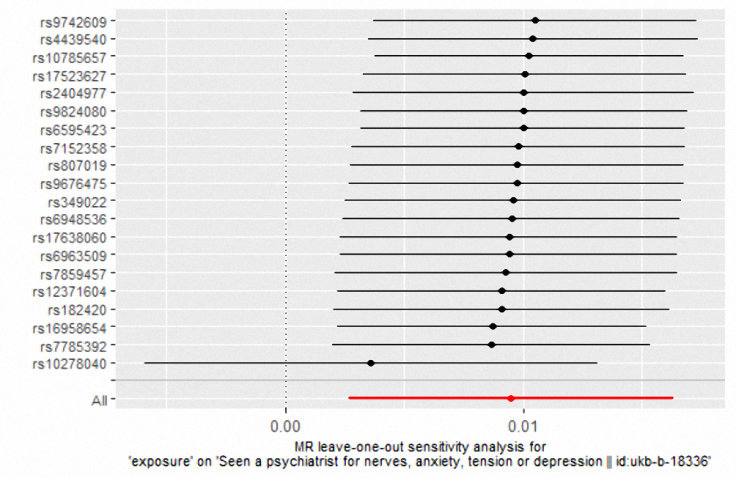


**Figure S4**:Leave-one-out plots for 1-linoleoylglycerophosphoethanolamine on anxiety disorders diagnosed by psychiatrists.


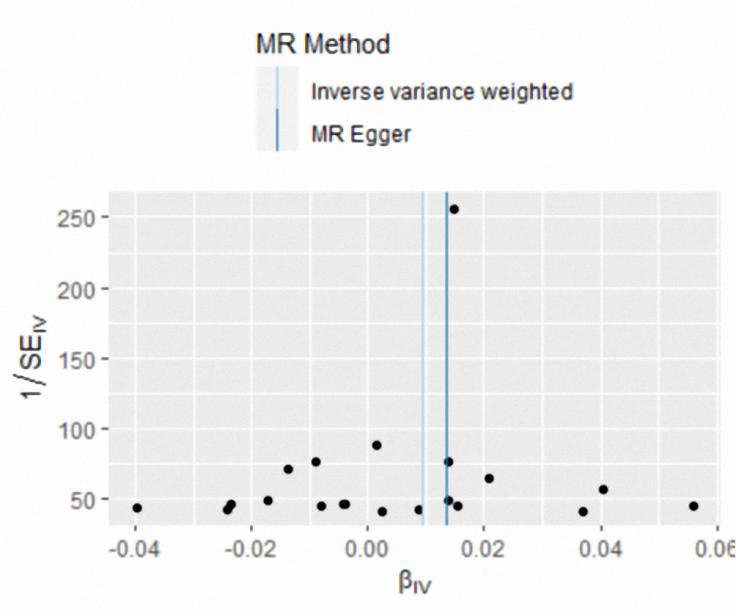


**Figure S5**:Funnel plots for 1-linoleoylglycerophosphoethanolamine on anxiety disorders diagnosed by psychiatrists.

**
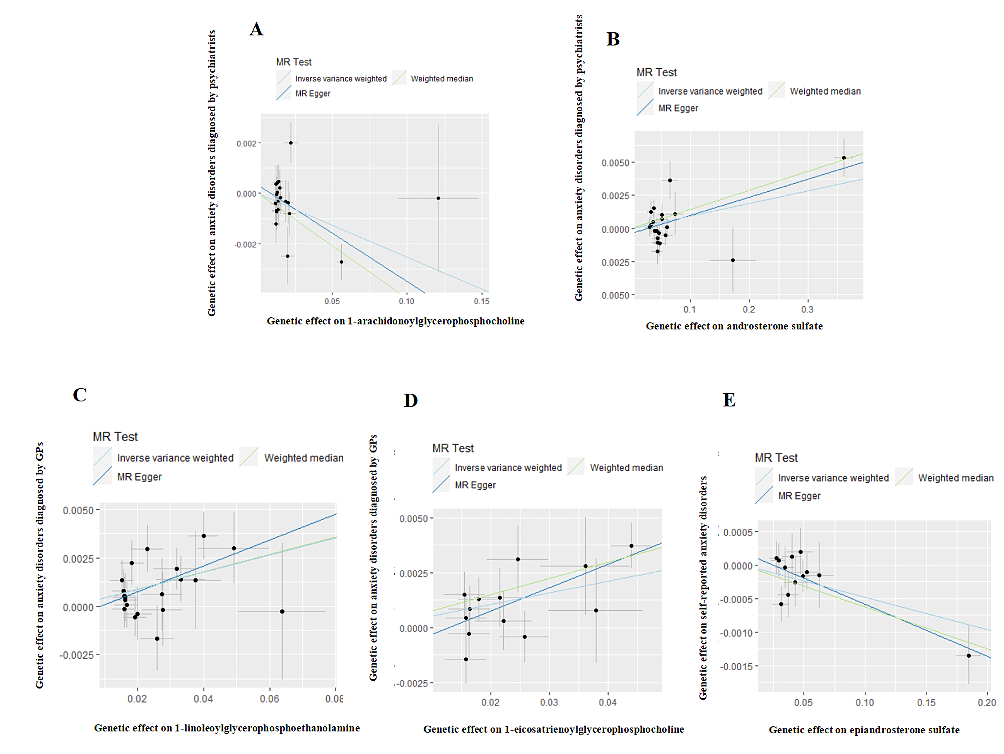
**

**Figure S6**: Scatter plots of the genetic association of six metabolites on the risk of anxiety disorders.1-arachidonoylglycerophosphocholine on anxiety disorders diagnosed by psychiatrists(A), androsterone sulfate on anxiety disorders diagnosed by psychiatrists(B), 1-linoleoylglycerophosphoethanolamine on anxiety disorders diagnosed by general practitioners(GPs) (C), 1-eicosatrienoylglycerophosphocholine on anxiety disorders diagnosed by general practitioners(GPs) (D), epiandrosterone sulfate on self-reported anxiety disorders(E).

**
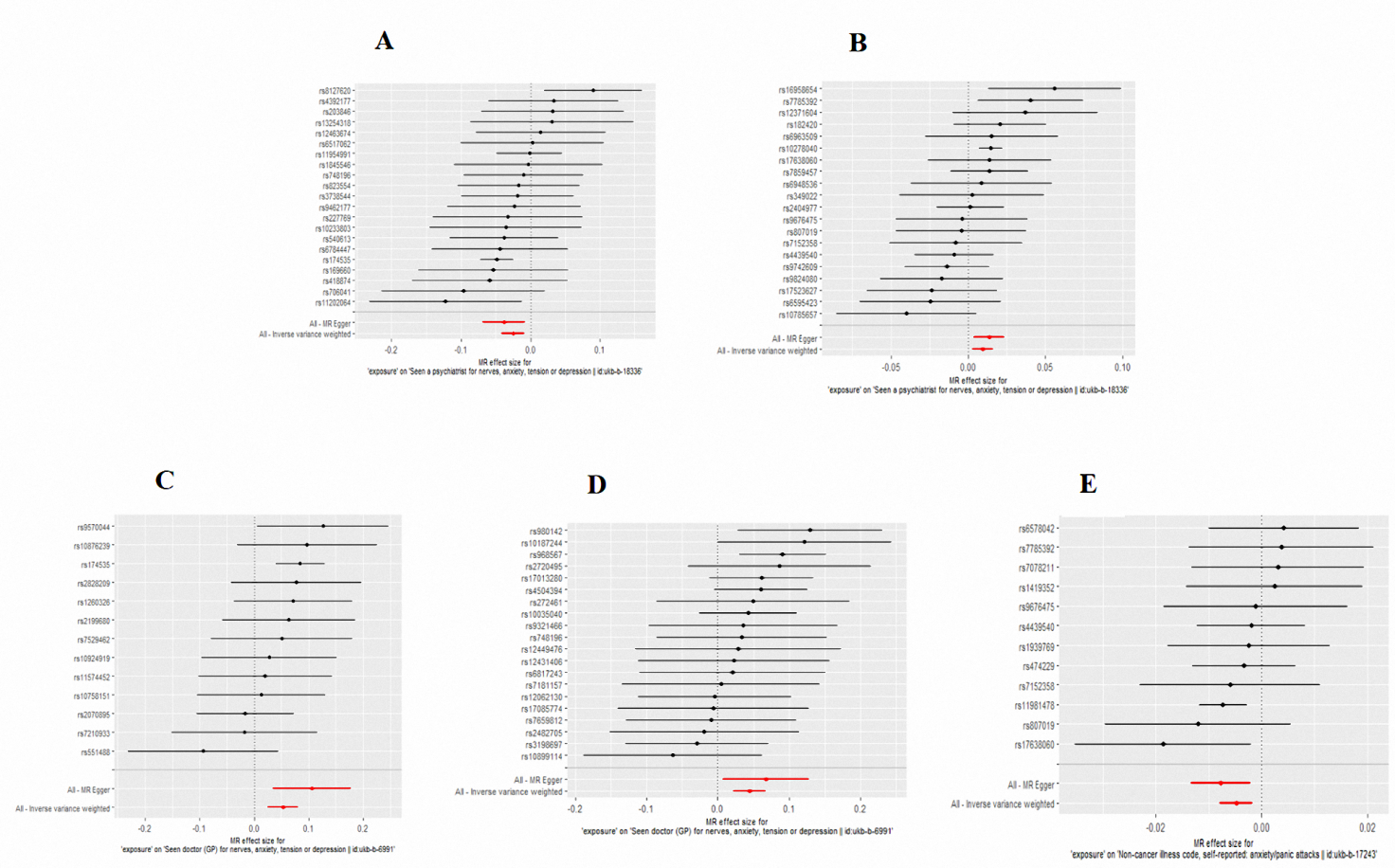
**

**Figure S7**:Forest plots for six potential metabolites on anxiety disorders.1-arachidonoylglycerophosphocholine on anxiety disorders diagnosed by psychiatrists(A), androsterone sulfate on anxiety disorders diagnosed by psychiatrists(B), 1-linoleoylglycerophosphoethanolamine on anxiety disorders diagnosed by general practitioners(GPs) (C),1-eicosatrienoylglycerophosphocholine on anxiety disorders diagnosed by general practitioners(GPs) (D), epiandrosterone sulfate on self-reported anxiety disorders(E).


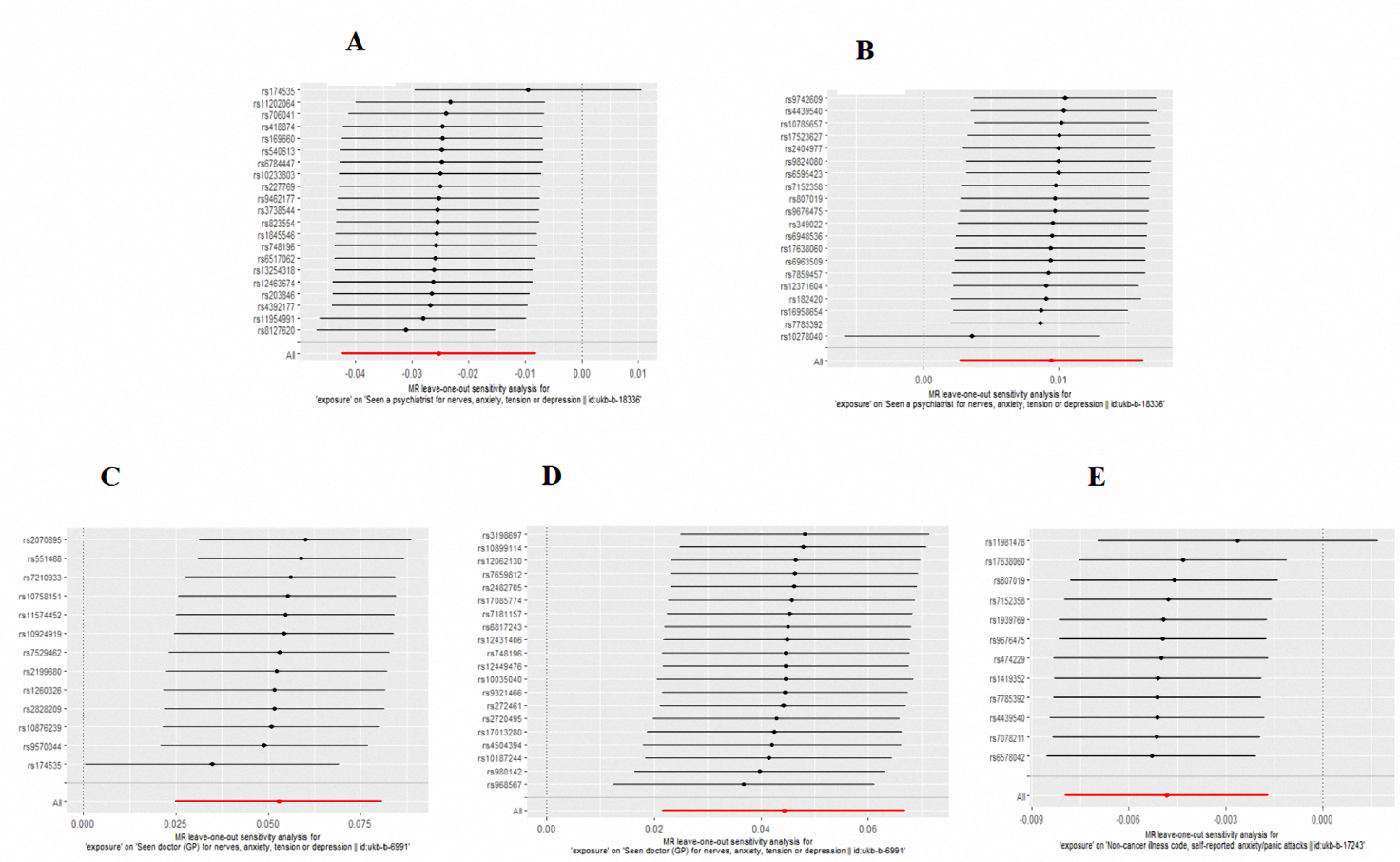


**Figure S8**:Leave-one-out plots for the six potential metabolites on anxiety disorders.1-arachidonoylglycerophosphocholine on anxiety disorders diagnosed by psychiatrists(A), androsterone sulfate on anxiety disorders diagnosed by psychiatrists(B), 1-linoleoylglycerophosphoethanolamine on anxiety disorders diagnosed by general practitioners(C), 1-eicosatrienoylglycerophosphocholine on anxiety disorders diagnosed by general practitioners(D), epiandrosterone sulfate on self-reported anxiety disorders(E).


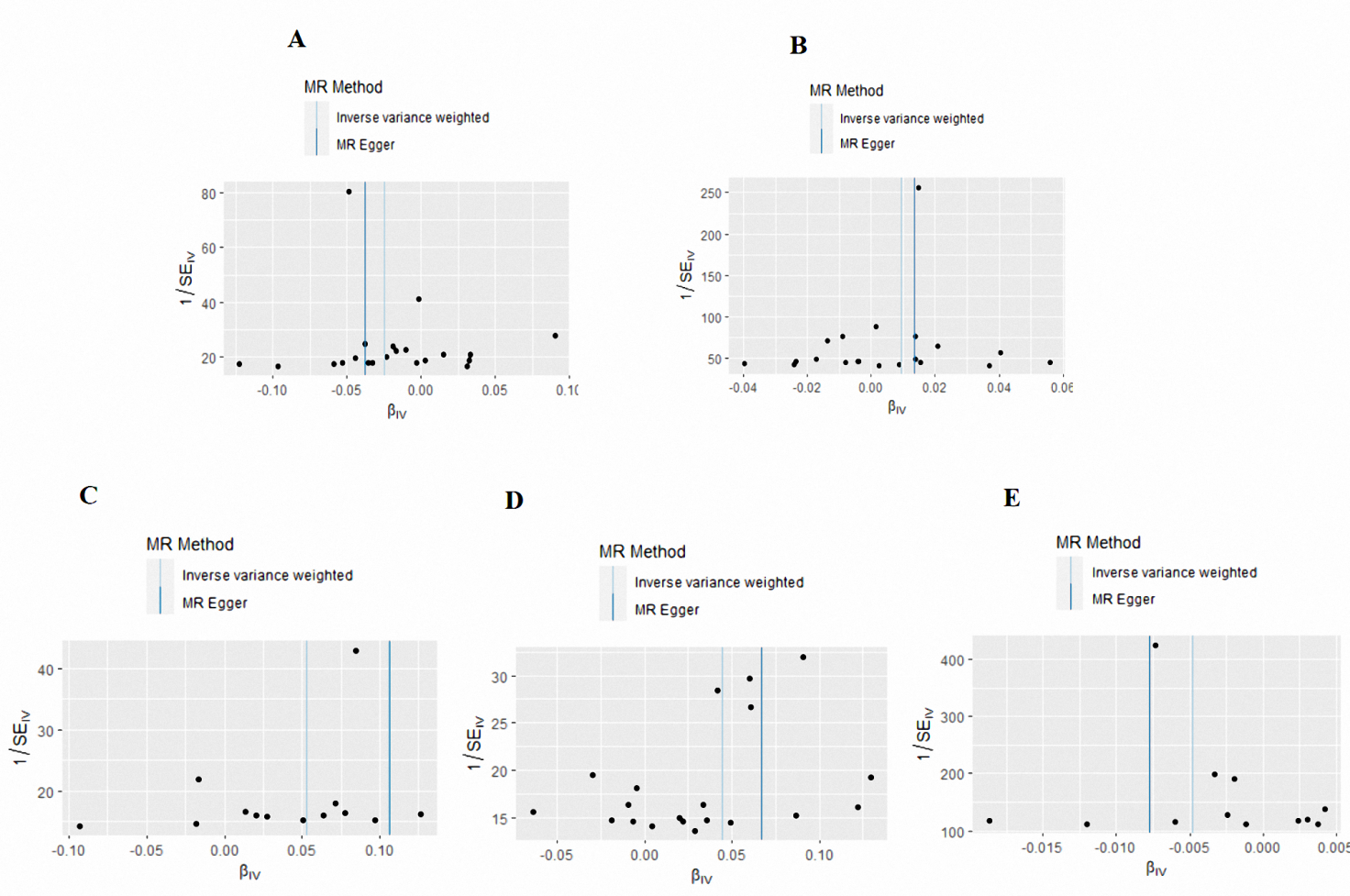


**Figure S9:**Funnel plots for six potential metabolites on anxiety disorders. 1-arachidonoylglycerophosphocholine on anxiety disorders diagnosed by psychiatrists(A), androsterone sulfate on anxiety disorders diagnosed by psychiatrists(B), 1-linoleoylglycerophosphoethanolamine on anxiety disorders diagnosed by general practitioners(C), 1-eicosatrienoylglycerophosphocholine on anxiety disorders diagnosed by general practitioners(D), epiandrosterone sulfate on self-reported anxiety disorders(E).

**
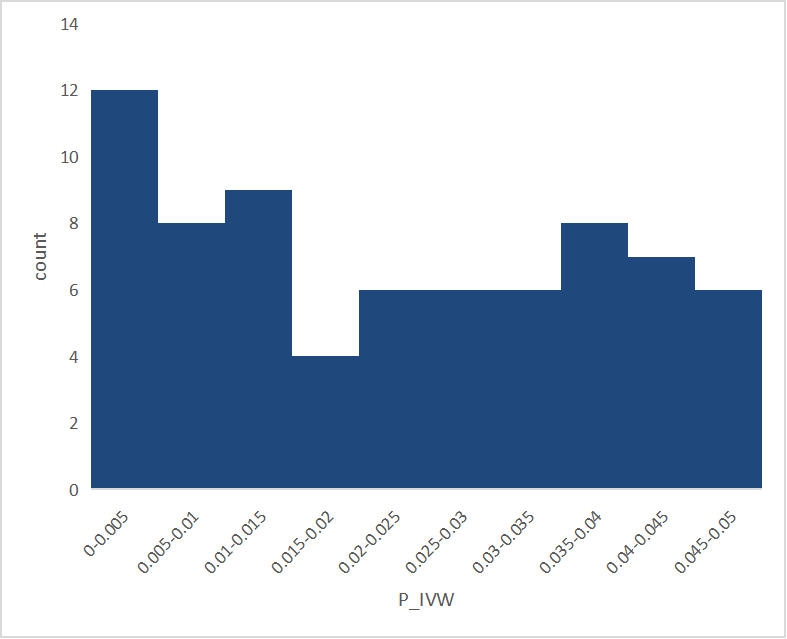
**

**Figure S10:** The p-value distribution of the P_IVW＜0.05 metabolites on anxiety disorder.
